# Risk factors analysis of endoscopy and TIPS in the treatment of secondary esophagogastric varicose bleeding with cirrhosis

**DOI:** 10.4314/ahs.v23i3.76

**Published:** 2023-09

**Authors:** Xiaoyan Liu, Yanli Wang, Lei Zheng, Jinzhao Zhu

**Affiliations:** Mengchao Hepatobiliary Hospital of Fujian Medical University, Fuzhou 350025, China

**Keywords:** Liver cirrhosis, esophagogastric varicose bleeding, Endoscopy, TIPS

## Abstract

**Objective:**

To analyse the risk factors of secondary hemorrhage and survival rate in cirrhotic patients with esophagogastric variceal rupture and to compare the efficacy and safety of endoscopic hemostasis and TIPS (transjugular intrahepatic portosystemic shunt).

**Methods:**

A total of 120 patients with secondary bleeding after endoscopic treatment of esophagogastric varicose bleeding with cirrhosis in our hospital during the past 3 years were retrospectively analysed. There were 65 males and 55 females, ranging in age from 49 to 74 years old, with an average of (59.5 ± 8.4) years old. The etiology, degree of varicose veins, bleeding location, hemostasis method, Infection, ascites, portal vein thrombosis or cancer thrombus, albumin, platelets, prothrombin activity, Child Pugh (Child-Pugh classification is a diagnostic criterion for liver reserve function) grade were compared in each group. The risk factors of treatment failure and analyse the survival time was analysed.

**Results:**

There were statistically significant differences in varicosis degree, infection, ascites, portal vein thrombosis or cancer thrombus, child Pugh grade, albumin and prothrombin activity between the failed Endoscopy group and the successful hemostasis group (P< 0.05). There were statistically significant differences in child Pugh grade, albumin and prothrombin activity between the failed TIPS treatment group and successful hemostasis group (P< 0.05). There was no significant difference in 1-year survival between the endoscopy group and the TIPS group.

**Conclusion:**

Severe varicose veins, infection, ascites, portal vein thrombosis or cancer thrombus, child pugh classification, albumin, and prothrombin activity were the major risk factors for failed secondary endoscopic therapy, child Pugh classification, albumin and prothrombin activity were the main risk factors for failure TIPS treatment. There is no significant difference in long-term survival between the two methods.

## Introduction

Esophagogastric varices bleeding is the end-stage manifestation of liver cirrhosis and portal hypertension caused by various reasons, and it is one of the high-risk factors leading to death of patients. Endoscopic hemostasis is one of the timely and effective methods for the treatment of esophagogastric variceal bleeding, recognized by current guidelines. TIPS is one of the key measures to reduce portal pressure in patients with liver cirrhosis by establishing a shunt channel in the liver parenchyma between the hepatic vein and the portal vein in a minimally invasive way 1. Both early transjugular intrahepatic portosystemic shunt (TIPS) and salvage TIPS can be used for the treatment of EGVB, and can also be used as a first-line regimen for the secondary prevention of EGVB. TIPS can better reduce portal pressure, and has a clear effect on EGVB 2. However, some patients may still experience rebleeding after endoscopic hemostasis. Bleeding, and the main factors of secondary bleeding and whether to continue to use endoscopic or TIPS treatment after bleeding, there is no clear conclusion at home and abroad.

The occurrence and development of esophagogastric varices, and how to choose the appropriate means for rebleeding to reduce the risk of rebleeding, thereby improving the curative effect and reducing the mortality rate, are clinical issues worthy of attention. This study retrospectively analysed the cases of liver cirrhosis with esophagogastric varices hemorrhage treated in our hospital in recent years. We analysed the risk factors of endoscopic and TIPS therapy for secondary bleeding in patients with cirrhotic esophagogastric variceal bleeding after endoscopic therapy, and compared the efficacy and safety of endoscopic hemostasis and TIPS therapy.

At present, there are few reports on the combination of TIPS and endoscopic hemostasis in the treatment of esophagogastric variceal bleeding in patients with liver cirrhosis. The purpose of this study is to predict the progression of the disease and evaluate the treatment risk for patients with multiple ruptured bleeding from esophagogastric varices.

## Materials and methods

### Ethics statement

All patients were informed of the necessity of treatment and possible complications before surgery, and signed informed consent for surgery. This study was approved by the Medical Ethics Committee of Mengchao Hepatobiliary Hospital of Fujian Medical University, Fuzhou 350025, China.

### Research subjects and groupings

Select patients with secondary bleeding from cirrhotic esophagogastric varices bleeding after endoscopic treatment in our hospital in the past 3 years. There were 65 males and 55 females, ranging in age from 49 to 74 years old, with average of (59.5 ± 8.4) years old. The cases were divided into 4 groups: the successful endoscopic treatment group, the failed endoscopic treatment group, the successful TIPS treatment group, the TIPS treatment failure group.

**Inclusion criteria:** (1) The patients meet the diagnostic criteria for liver cirrhosis; (2) The history of esophagogastric varices rupture has occurred in the past. Bleeding and treated with endoscopic band ligation, sclerotherapy, or tissue glue injection.

**Exclusion criteria:** (1) Patients with gastrointestinal bleeding caused by other parts or other reasons; (2) Patients with blood system diseases; (3) Patients with severe cardiopulmonary diseases, multiple organ failure, and patients who cannot cooperate with endoscopy or interventional therapy.

### Treatment methods

Before surgery, patients with severe anemia were treated with blood transfusion to maintain stable vital signs. Biochemical, imaging examinations, and Child Pugh scoring system were used to evaluate liver reserve function. Portal vein CT angiography (CT angiography, CTA) was used to evaluate the portal vein to see if there was thrombosis, to determine whether there were collateral blood vessels, and to evaluate the shunt status of gastric varices. The postoperative hemostasis success rate and bleeding cessation time of the four groups of patients were observed, and the success of hemostasis was based on whether emergency endoscopic or interventional therapy was required within 24 hours after surgery, and the time of stool turning yellow was used as the successful hemostasis time.

The degree of endoscopic varicose veins, the changes of liver function Child-Pugh classification, the rebleeding rate of esophagogastric varices and the 1-year survival rate were observed at 3 months, 6 months and 12 months after operation. The LDRF (L represents the location of the varicose veins, D represents the largest diameter of the varicose veins observed, and Rf represents the risk of variceal bleeding) classification standard is used for staging of varicose veins under endoscopy 3. Esophagogastric variceal hemorrhage was determined by endoscopic findings of active variceal bleeding (ejection-like bleeding, oozing), thrombus head formation, and the presence of obvious variceal veins but no other bleeding lesions were found 4.

All patients were treated with standard endoscopic band ligation, sclerotherapy and tissue glue injection according to the location and degree of varicose veins 5. TIPS was performed in the intervention room, the right femoral artery was punctured using Seldinger's technique, and a 6F catheter sheath was placed. The catheter was supers elected into the superior mesenteric artery, eldinger's technique was used to puncture the right internal jugular vein, and a RUPS 100 long sheath was inserted to super select it into the right hepatic vein. Angiography of the superior mesenteric artery showed the main portal vein, left and right branches. The right branch of the portal vein was successfully punctured with a puncture needle (RUPS 100), and a 4F single-curved catheter was sent to the superior mesenteric vein. Splenic venography showed that the portal vein and splenic vein issued multiple varicose vessels to supply the esophagus and gastric fundus varices. The Amplatz guide wire was sent to the superior mesenteric vein, and the RUPS-100 sheath was sent to the main portal vein. The catheter combined with the microcatheter was supers elected to the varicose veins from the portal vein and splenic vein.

The gold-marked catheter locates the stent placement position and length, adjusts the position of the Viator to the appropriate diameter, and releases the stent-graft, and then expands the stent with a balloon, and then the stent was expanded with a balloon. After the stent was released, portal venography was performed again and the portal vein pressure gradient was re-measured to determine whether the shunt was successful. Intraoperative intravenous administration of 3000U heparin sodium was given. The patient was supine for 1 day after the operation, and a low-protein diet was given. Routine treatment was given to prevent infection, acid suppression, and anti-hepatic encephalopathy. Patients without obvious coagulation dysfunction were subcutaneously injected with 3000 u of low molecular weight heparin, 2 times a day, and changed to oral administration after 1 week.

### Follow-up and efficacy judgment

The guidelines for the prevention and treatment of liver cirrhosis with EGVB propose that the symptoms of hematemesis and gastrointestinal bleeding occur again within 24 hours after surgery, and the hemoglobin progressively decreases by more than 30 g/mL without blood transfusion. The systolic blood pressure decreases by more than 20 mL of mercury. Symptoms of hemorrhagic shock, such as column or heart rate increase of more than 20 beats/min, were judged to have failed hemostatic therapy.

### Observation indicators

Data of patients were collected, including gender, age, record its etiology, degree of varicose veins, bleeding location, treatment method, presence or absence of co-infection, ascites, portal vein thrombosis or tumor thrombus, albumin, platelets, and prothrombin activities. Assess the patient's Child Pugh grade. Statistical factors influencing treatment failure. Compare their survival rates.

### Statistical analysis

SPSS 20.0 software was used for statistical analysis. The normally distributed measurement data is expressed as mean±SD, and the test comparison is made by t-test; the measurement data with skewed distribution is expressed as the median (lower quartile, upper quartile), and the rank sum is used for comparison test. The enumeration data were expressed as the number of cases and percentages, and the comparison was performed using the chi-square test. P<0.05 was considered statistically significant, and the survival curve was tested by Log-Rank test.

## Results

Comparison of general data between the successful and failed groups of endoscopic and TIPS treatment. There was no significant difference in the general data of gender and age in each group (all P>0.05) (Table 1; Table 2).

**Table 1:** Comparison of general data of patients in the successful and failed endoscopic treatment groups

Group	Endoscopic treatment success group	Endoscopic treatment failure group	χ^2^/t	P value
Gender				
Male	31	8		
Female	14	7	χ^2^=1.197	p= 0.274
Average age	55.2±10.2	58.6±11.0	t=1.103	p= 0.274

**Table 2:** Comparison of general data of patients in the successful and failed TIPS treatment groups

Group	Endoscopic treatment success group	Endoscopic treatment failure group	χ^2^/t	P value
Gender				
Male	37	7		
Female	15	1	χ^2^=0.947	p= 0.330
Average age	54.8±11.6	59.2±12.6	t=0.989	p = 0.327

### Analysis of the influencing factors of the failure of endoscopic hemostatic therapy

The etiology, degree of varicose veins, bleeding location, treatment method, co-infection, ascites, portal vein thrombosis or tumor thrombus, albumin, platelets, thrombin were compared between the two groups of patients. Original activity, and Child Pugh classification (Table 3). The severity of varicose veins, co-infection, ascites, portal vein thrombosis or tumor thrombus, Child Pugh grade C in the failed endoscopic treatment group were higher than those in the successful hemostasis group, and the activities of albumin and prothrombin were lower than those in the successful hemostasis group P<0.05, the difference was statistically significant.

**Table 3:** Analysis of influencing factors of endoscopic therapy

Factor	Endoscopic treatment success group	Endoscopic treatment failure group	χ^2^/t	P value
**Cause**				
Post-hepatitis B/C cirrhosis group	34	9		
Alcoholic cirrhosis group	5	3		
Autoimmune hepatitis group	3	2		
Other	3	1	χ^2^=1.647	p=0.649
**Degree of varicose veins**				
Severe	28	14		
Moderate	17	1	χ^2^=5.185	p=0.023
**Bleeding location**				
Middle esophagus	7	1		
Lower esophagus	27	7		
Cardia	8	5		
Fundus	2	2	χ^2^=3.210	p=0.360
**Treatment**				
EVL Treatment	31	9		
Tissue glue injection	14	6	χ^2^=0.400	p=0.527
Coinfection	21	12		
No co-infection	24	3	χ^2^=5.051	p=0.025
With ascites	30	14		
No ascites	15	1	χ^2^=4.091	p=0.043
Portal vein thrombosis or tumor thrombus	14	10		
No portal vein thrombosis or tumor thrombus	31	5	χ^2^=5.926	p=0.015
**Blood index**				
Median (lower quartile, upper quartile)				
Albumin	31.0 (28.0,33.5)	27.0 (25.0,29.0)	z =-2.455	p=0.014
Platelet count	78.0 (50.0,103)	68.0 (66.0,101.0)	z =-0.572	p=0.567
Prothrombin activity	63.0 (55.5,81.5)	44..0 (38.0,64.0)	z =-2.486	p=0.013
**Child Pugh**				
Class A	21	2		
Class B	12	3		
Class C	12	10	χ^2^=8.370	p=0.015

### Analysis of the influencing factors of TIPS hemo-static treatment failure

The etiology, degree of varicose veins, bleeding location, presence or absence of co-infection, ascites, portal vein thrombosis or tumor thrombus, albumin, platelets, prothrombin activity, Child Pugh classification, the proportion of Child Pugh classification C in the TIPS hemostasis failure group was higher than that in the successful hemostasis group, and the activities of albumin and prothrombin were lower than those in the successful hemostasis group, P<0.05, and the differences were statistically significant (Table 4).

**Table 4:** Analysis of Influencing Factors of TIPS Treatment

Factor	Endoscopic treatment success group	Endoscopic treatment failure group	χ^2^/t	P value
**Cause**				
Post-hepatitis B/C cirrhosis group	39	4		
Alcoholic cirrhosis group	9	4		
Autoimmune hepatiti group	4	0		
Other	0	0	χ^2^=4.640	p = 0.098
**Degree of varicose veins**				
Severe	48	7		
Moderate	4	1	χ^2^=0.210	p = 0.647
**Bleeding location**				
Middle esophagus	7	0		
Lower esophagus	42	6		
Cardia	2	1		
Fundus	1	1	χ^2^=4.471	p = 0.215
Coinfection	30	7		
No co-infection	22	1	χ^2^=2.606	p = 0.106
With ascites	32	7		
No ascites	20	1	χ^2^=2.054	p = 0.152
Portal vein thrombosis or tumor thrombus	16	4		
No portal vein thrombosis or tumor thrombus	36	4	χ^2^=1.154	p = 0.283
**Blood index**				
Albumin			z = -1.798	p = 0.072
Platelet count			z = -1.120	p = 0.263
Prothrombin activity			z =-3.026	p = 0.002
**Child Pugh**				
Class A				
Class B				
Class C			χ^2^=7.582	p = 0.023

### Long-term survival

The successful cases were followed up for 1 year, 5 cases (11.11%) in the endoscopic group died, and the survival rate was 88.89%; 3 cases (5.77%) in the TIPS group died, and the survival rate was 94.23%; Kaplan-Meier Survival curve, the results suggest that P = 0.413> 0.05, no statistical difference, indicating that there is no significant difference in long-term survival between the two groups.

## Discussion

Esophagogastric variceal bleeding is a common acute and critical illness in digestive system diseases 5. The mortality rate of such patients can reach 25%-50% within 1 week without treatment and intervention, and the mortality rate within 1 year is as high as 70% 6,7. Endoscopic hemostasis has been the main method for the treatment of first bleeding from esophagogastric vein rupture 8, but there are still 10%-20% of patients with first successful hemostasis who experience secondary bleeding, and secondary hemostasis failure is more likely to lead to death 9. The evaluation of the first hemostatic treatment has been widely reported in many domestic and foreign literatures, but the treatment of hemostasis in patients with rebleeding is rarely evaluated. In clinical work, due to the existence of many contraindications to surgery, and due to various reasons, such as patient compliance and economic conditions, some patients did not choose surgery and liver transplantation. Because of repeated bleeding, multiple endoscopic hemostasis and TIPS were performed.

In the present study, by exploring the risk factors related to the failure of secondary hemostatic therapy, we analysed the success rate of endoscopic and TIPS therapy, and evaluated the secondary hemostatic therapy in patients with esophagogastric varices bleeding, in order to provide effective treatment for patients with esophagogastric varices bleeding. We retrospectively analysed and compared the etiology, varicose veins, bleeding location, co-infection, ascites, portal vein thrombosis or tumor thrombus, albumin, platelets, prothrombin activity, and Child Pugh grade. Factor analysis showed that the severity of varicose veins, co-infection, ascites, portal vein thrombus or tumor thrombus, Child Pugh grade C in the failed endoscopic treatment group were higher than those in the successful hemostasis group, and the activities of albumin and prothrombin were lower than those in the successful hemostasis group. The proportion of Child Pugh grade C in the TIPS hemostasis failure group was higher than that in the successful hemostasis group, and the activities of albumin and prothrombin were lower than those in the successful hemostasis group. It is suggested that these factors may be the risk factors for the failure of hemostatic treatment for secondary bleeding in patients with cirrhotic esophagogastric varices bleeding.

Some studies have shown that the degree of esophageal varices and portal vein pressure gradient are risk factors for predicting esophagogastric variceal bleeding, and the risk of variceal bleeding increases when the portal vein pressure gradient is greater than 12 mmHg 10. Abraldes showed that the degree of esophageal varices in patients with Child Pugh C-level liver function was significantly greater than that in A-level patients, indicating that esophageal varices bleeding is closely related to liver fuction status. However, the bleeding risk of Child Pugh A patients with severe varices and red sign was significantly higher than that of C patients, indicating that the severity of esophageal varices is one of the important factors affecting bleeding 11. Hino found that the risk of variceal bleeding is higher when the diameter of gastric coronary vein is greater than 6.5 mm, which is similar to the results of this study 12.

Other studies have also shown that ascites and portal vein emboli are also high-risk factors for esophageal variceal bleeding 11. The report of Noronha Ferreira mentioned that the formation of portal vein emboli hinders the return of portal blood, aggravates portal hypertension, and further increases the risk of variceal bleeding 13. This study also found an independent risk factor for infection hemostasis failure in the endoscopic group. Considering that the presence of infection affects the healing of wounds after ligation, it is more likely to have bleeding risk in the shedding period, resulting in operation failure. In addition, hypoalbuminemia, decreased prothrombin activity, and the proportion of high Child Pugh grade C are also common risk factors for endoscopic and TIPS treatment failure. Considering them reflect the patient's liver reserve function and disease severity, coagulation function and Hemostatic therapy is more likely to fail in patients with poor hepatic reserve. Comparing the two treatment methods, the author believes that TIPS has more advantages in long-term hemostasis. During the operation of endoscopic therapy, only the obvious varicose esophageal vein can be ligated or injected with tissue glue. However, portal hypertension still persists, and new variceal vein branches can be formed again, causing another bleeding.

It is difficult to solve the portal hypertension. New varicose vein branches are formed again, causing another hemorrhage, and it is difficult to solve the problem of portal hypertension. TIPS improves the degree of varicose veins, relieves the pressure of the portal vein, reduces the risk of rebleeding, and reduces the pressure of the portal vein from the root, and has achieved good results in the prevention and treatment of rebleeding. It has gradually replaced surgical shunting and cutting. Surgery has become the main method for the treatment of bleeding complications of portal hypertension.

The treatment may lead to other complications such as hepatic encephalopathy and liver failure 15, 16. Analysis of the reasons shows that a large amount of portal blood is directly shunted into the vena cava, causing ischemia and atrophy of the liver, inducing or aggravating the decline of liver function, resulting in liver function failure. In addition, shunt failure is also a common complication that affects the long-term efficacy of TIPS, when stent stenosis or blockage occurs, treatment failure may occur, leading to recurrence of portal hypertension, and thus the risk of rebleeding. Therefore, the prevention and treatment of complications is the key to the efficacy of TIPS. In terms of long-term survival rates, the survival rates of patients in the endoscopic and TIPS treatment groups were 88.89% and 94.23%, respectively. There was no significant difference in the Log-rank test (P>0.05), indicating that early TIPS did not improve the survival rate of patients with liver cirrhosis. We believe that neither endoscopic nor TIPS therapy can prevent the progression of decompensated cirrhosis, which is the same as the findings of Holster 17.

In conclusion, when the above risk factors are found, it is necessary to inform patients of the efficacy, risks and complications of secondary hemostasis endoscopy and TIPS treatment, make predictions in advance, and make emergency and remedial plans. We need to maximize the success rate of hemostasis, improve the prognosis of patients with liver cirrhosis and esophagogastric varices bleeding, and improve the quality of life.

## Figures and Tables

**Figure 1:**
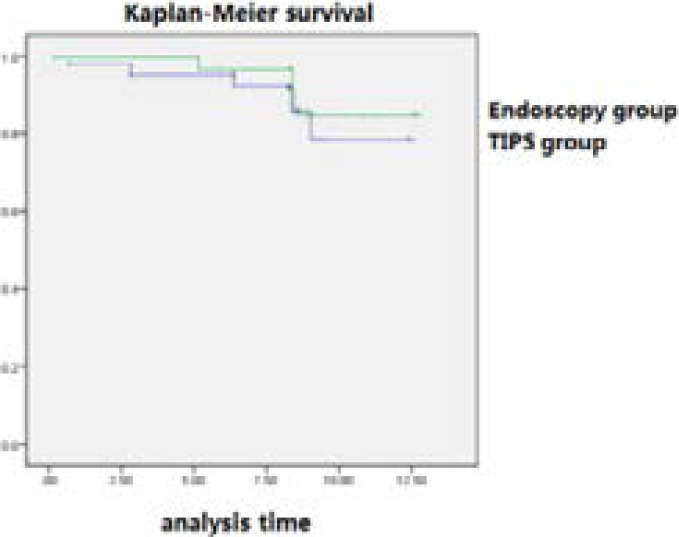
Survival curves of endoscopy group and TIPS group
